# Supplementary material for: Immunosuppressive Tumor Microenvironment of Osteosarcoma
Source: Cancers (Basel). 2025 Jun 24;17(13):2117. doi: 10.3390/cancers17132117 (PMC12248827; doi:10.3390/cancers17132117)
Supplement: Supplementary file 1 [file cancers-17-02117-s001.zip › Supplementary Figure S2 - inferCNV.pdf]

001_BX_COM

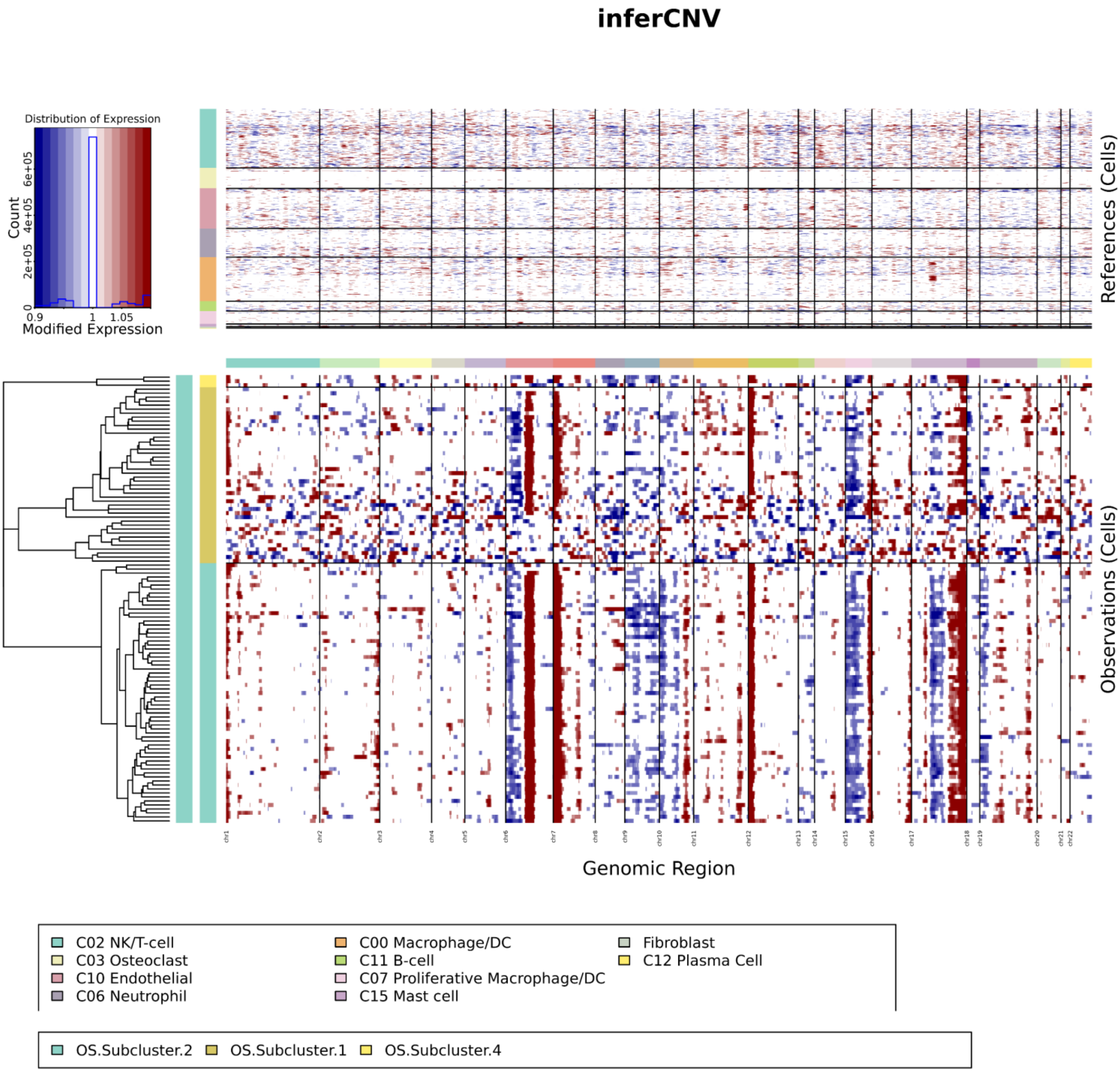
inferCNV
Distribution of Expression
Count
0
2e+05
4e+05
6e+05
0.9
1
1.05
Modified Expression
References (Cells)
Observations (Cells)
chr1 chr2 chr3 chr4 chr5 chr6 chr7 chr8 chr9 chr10 chr11 chr12 chr13 chr14 chr15 chr16 chr17 chr18 chr19 chr20 chr21 chr22
Genomic Region
C02 NK/T-cell
C03 Osteoclast
C10 Endothelial
C06 Neutrophil
C00 Macrophage/DC
C11 B-cell
C07 Proliferative Macrophage/DC
C15 Mast cell
Fibroblast
C12 Plasma Cell
OS.Subcluster.2
OS.Subcluster.1
OS.Subcluster.4

003_BX_COM

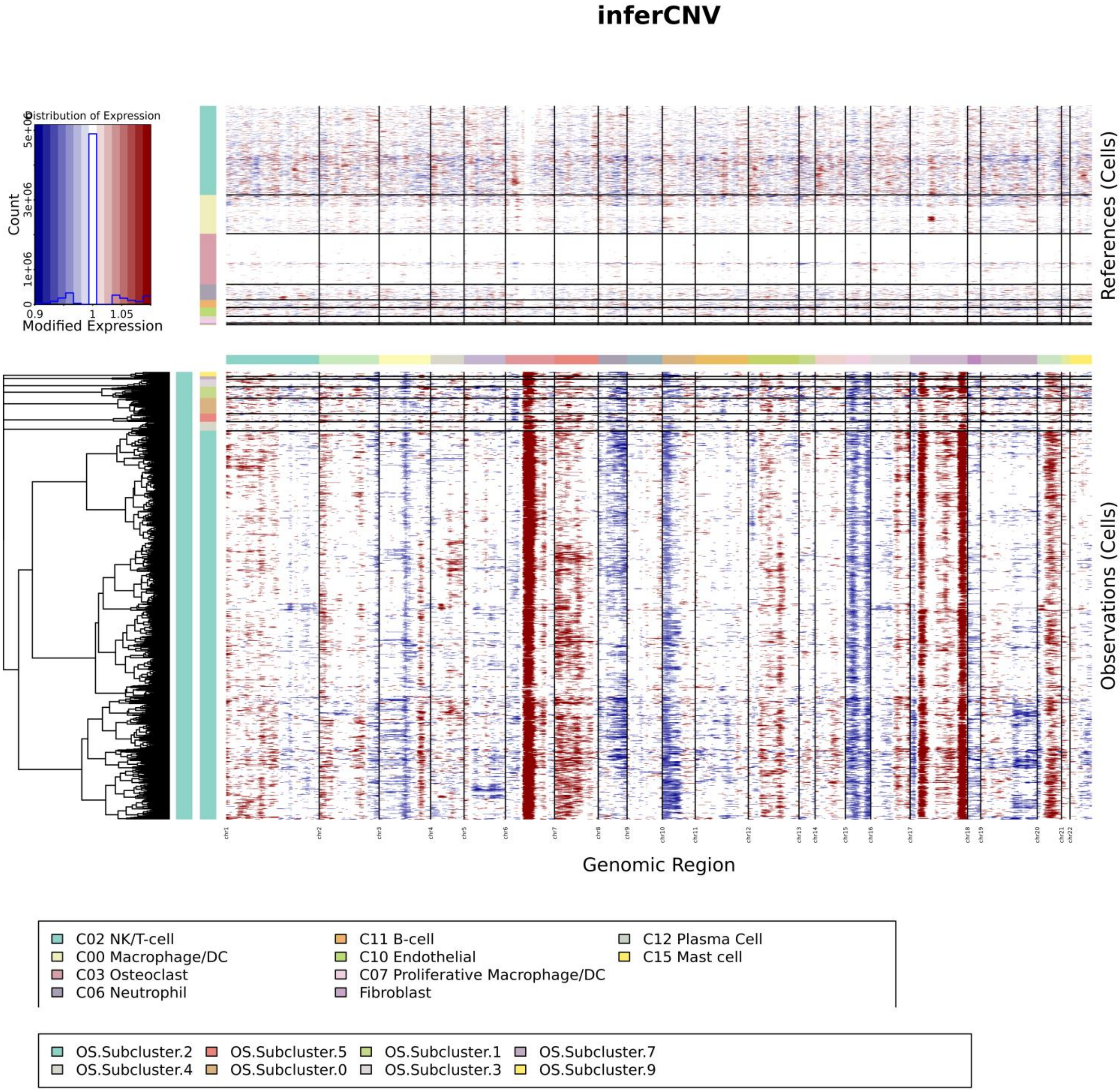
inferCNV
Distribution of Expression
Count
5e+06
3e+06
1e+06
0
0.9
1
1.05
Modified Expression
References (Cells)
Observations (Cells)
chr1
chr2
chr3
chr4
chr5
chr6
chr7
chr8
chr9
chr10
chr11
chr12
chr13
chr14
chr15
chr16
chr17
chr18
chr19
chr20
chr21
chr22
Genomic Region
C02 NK/T-cell
C00 Macrophage/DC
C03 Osteoclast
C06 Neutrophil
C11 B-cell
C10 Endothelial
C07 Proliferative Macrophage/DC
Fibroblast
C12 Plasma Cell
C15 Mast cell
OS.Subcluster.2
OS.Subcluster.4
OS.Subcluster.5
OS.Subcluster.0
OS.Subcluster.1
OS.Subcluster.3
OS.Subcluster.7
OS.Subcluster.9

010_BX_COM

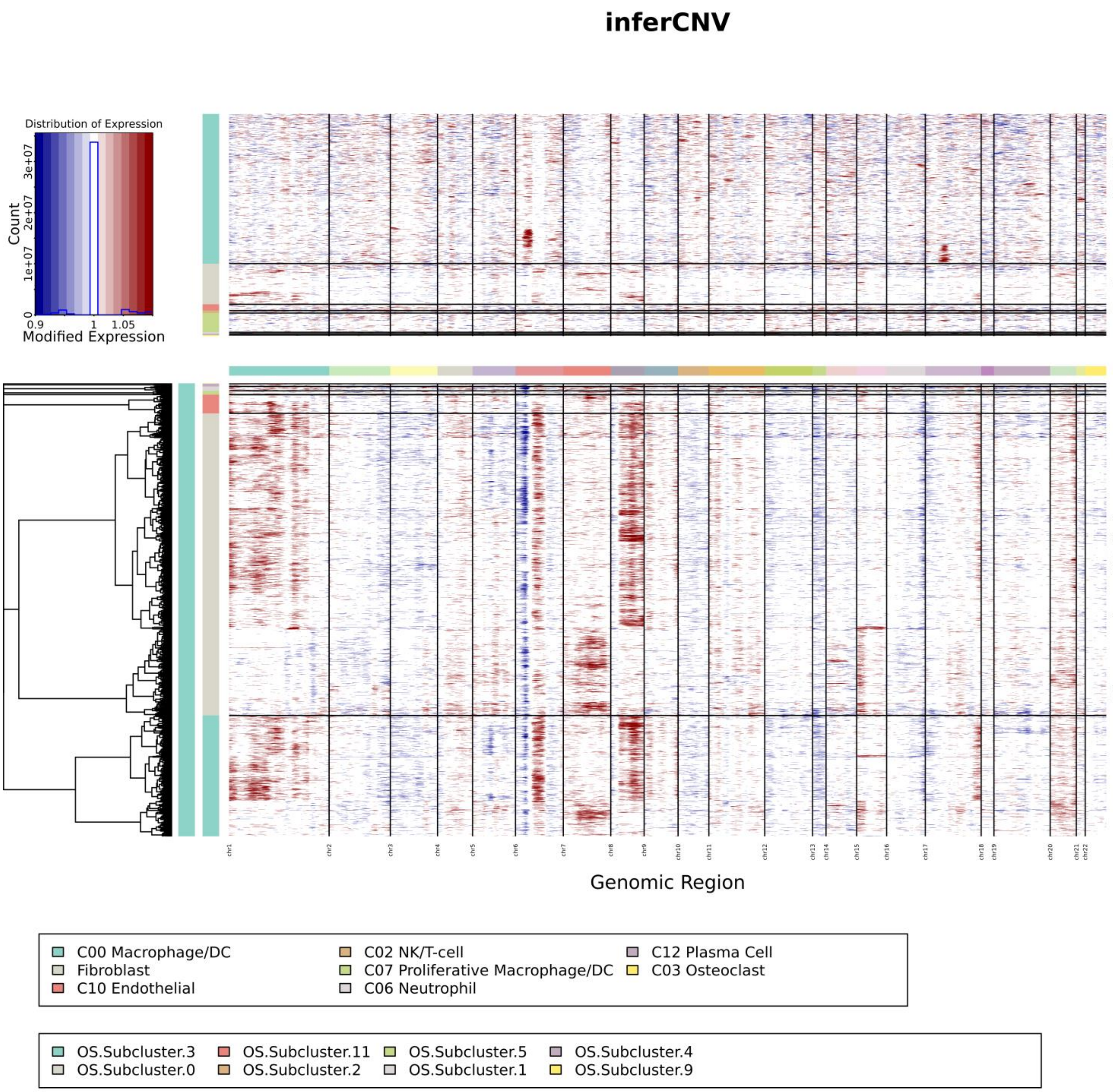
inferCNV
Distribution of Expression
Count
Modified Expression
References (Cells)
Observations (Cells)
Genomic Region
chr1 chr2 chr3 chr4 chr5 chr6 chr7 chr8 chr9 chr10 chr11 chr12 chr13 chr14 chr15 chr16 chr17 chr18 chr19 chr20 chr21 chr22
C00 Macrophage/DC
Fibroblast
C10 Endothelial
C02 NK/T-cell
C07 Proliferative Macrophage/DC
C06 Neutrophil
C12 Plasma Cell
C03 Osteoclast
OS.Subcluster.3
OS.Subcluster.0
OS.Subcluster.11
OS.Subcluster.2
OS.Subcluster.5
OS.Subcluster.1
OS.Subcluster.4
OS.Subcluster.9

OS.C.21.BX.OCT

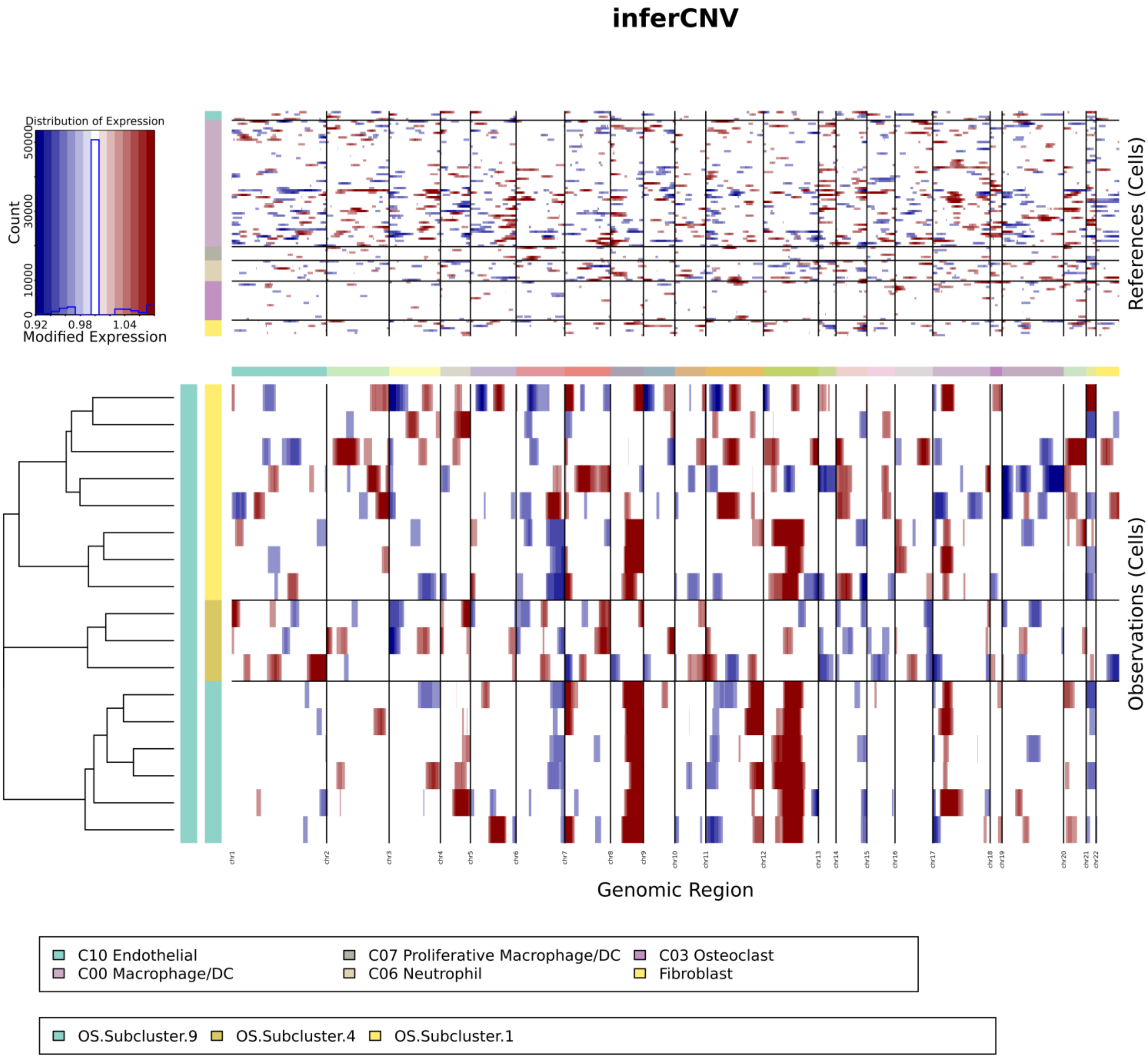
inferCNV
Distribution of Expression
Count
0
10000
30000
50000
0.92
0.98
1.04
Modified Expression
References (Cells)
Observations (Cells)
chr1
chr2
chr3
chr4
chr5
chr6
chr7
chr8
chr9
chr10
chr11
chr12
chr13
chr14
chr15
chr16
chr17
chr18
chr19
chr20
chr21
chr22
Genomic Region
C10 Endothelial
C00 Macrophage/DC
C07 Proliferative Macrophage/DC
C06 Neutrophil
C03 Osteoclast
Fibroblast
OS.Subcluster.9
OS.Subcluster.4
OS.Subcluster.1

OS.C.32.BX

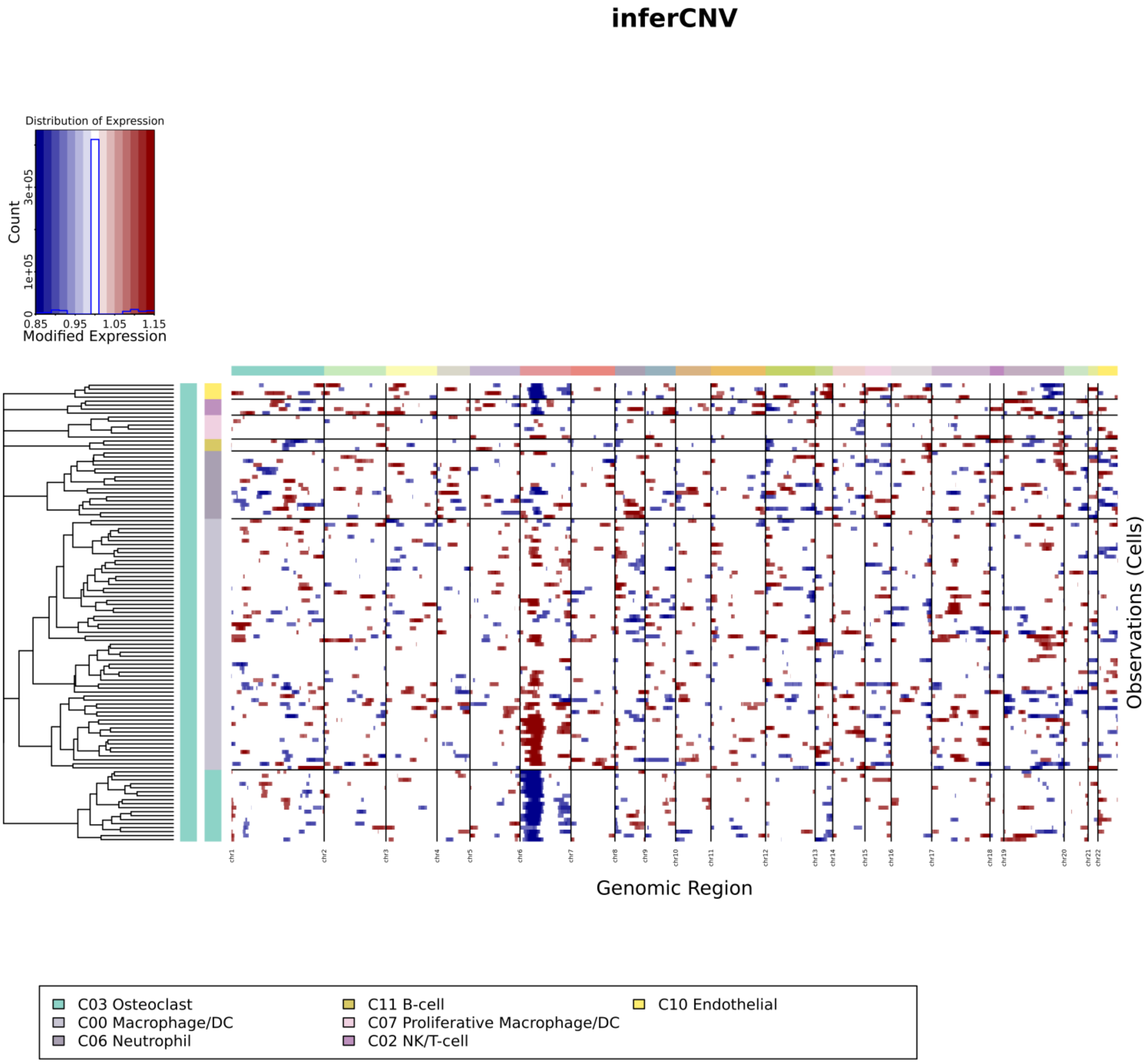
inferCNV
Distribution of Expression
Count
0
1e+05
3e+05
0.85
0.95
1.05
1.15
Modified Expression
chr1
chr2
chr3
chr4
chr5
chr6
chr7
chr8
chr9
chr10
chr11
chr12
chr13
chr14
chr15
chr16
chr17
chr18
chr19
chr20
chr21
chr22
Genomic Region
Observations (Cells)
C03 Osteoclast
C00 Macrophage/DC
C06 Neutrophil
C11 B-cell
C07 Proliferative Macrophage/DC
C02 NK/T-cell
C10 Endothelial

OS.C.33.BX

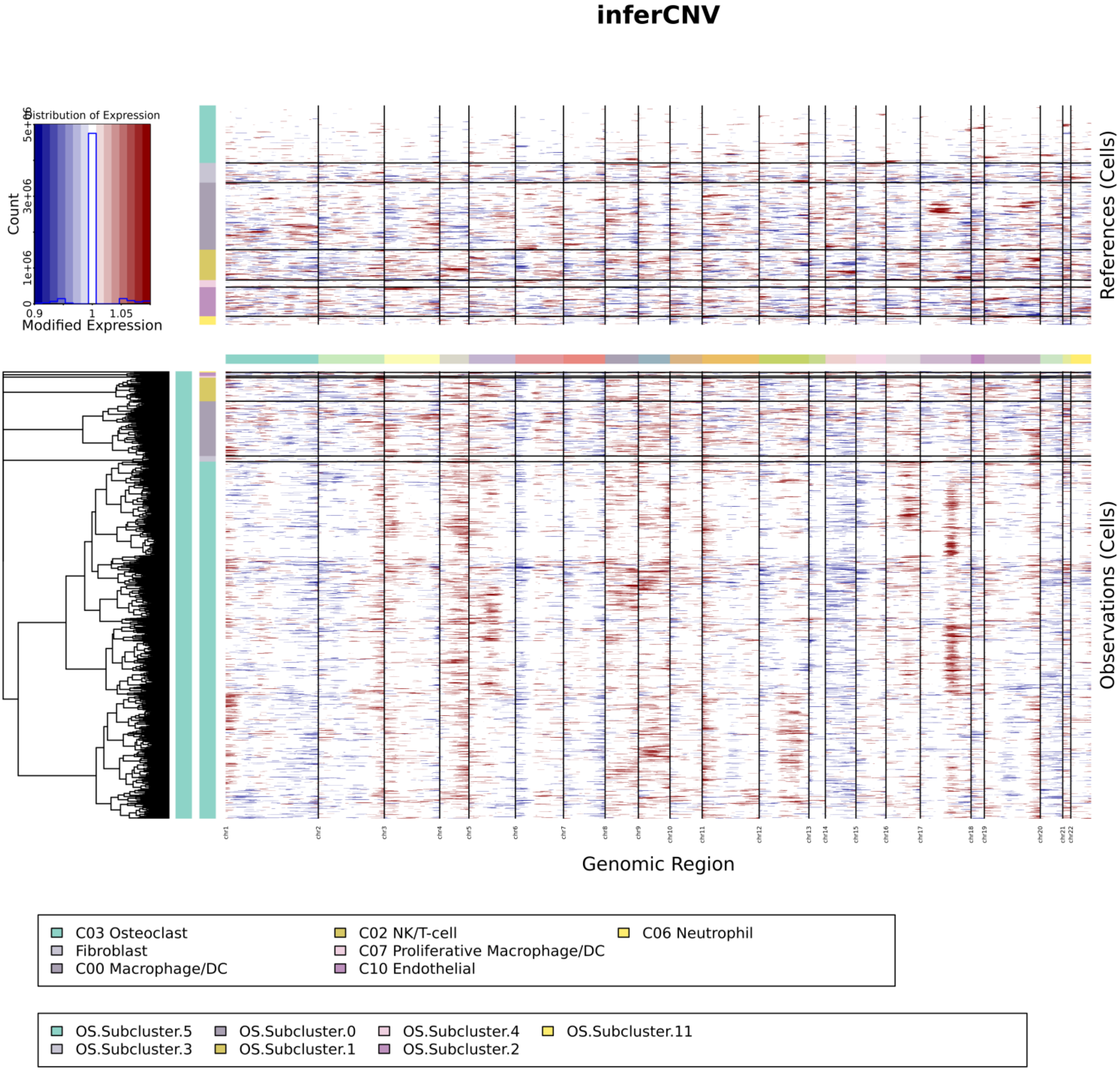
inferCNV
Distribution of Expression
Count
0
1e+06
3e+06
5e+06
0.9
1
1.05
Modified Expression
References (Cells)
Observations (Cells)
chr1
chr2
chr3
chr4
chr5
chr6
chr7
chr8
chr9
chr10
chr11
chr12
chr13
chr14
chr15
chr16
chr17
chr18
chr19
chr20
chr21
chr22
Genomic Region
C03 Osteoclast
Fibroblast
C00 Macrophage/DC
C02 NK/T-cell
C07 Proliferative Macrophage/DC
C10 Endothelial
C06 Neutrophil
OS.Subcluster.5
OS.Subcluster.3
OS.Subcluster.0
OS.Subcluster.1
OS.Subcluster.4
OS.Subcluster.2
OS.Subcluster.11

OS1

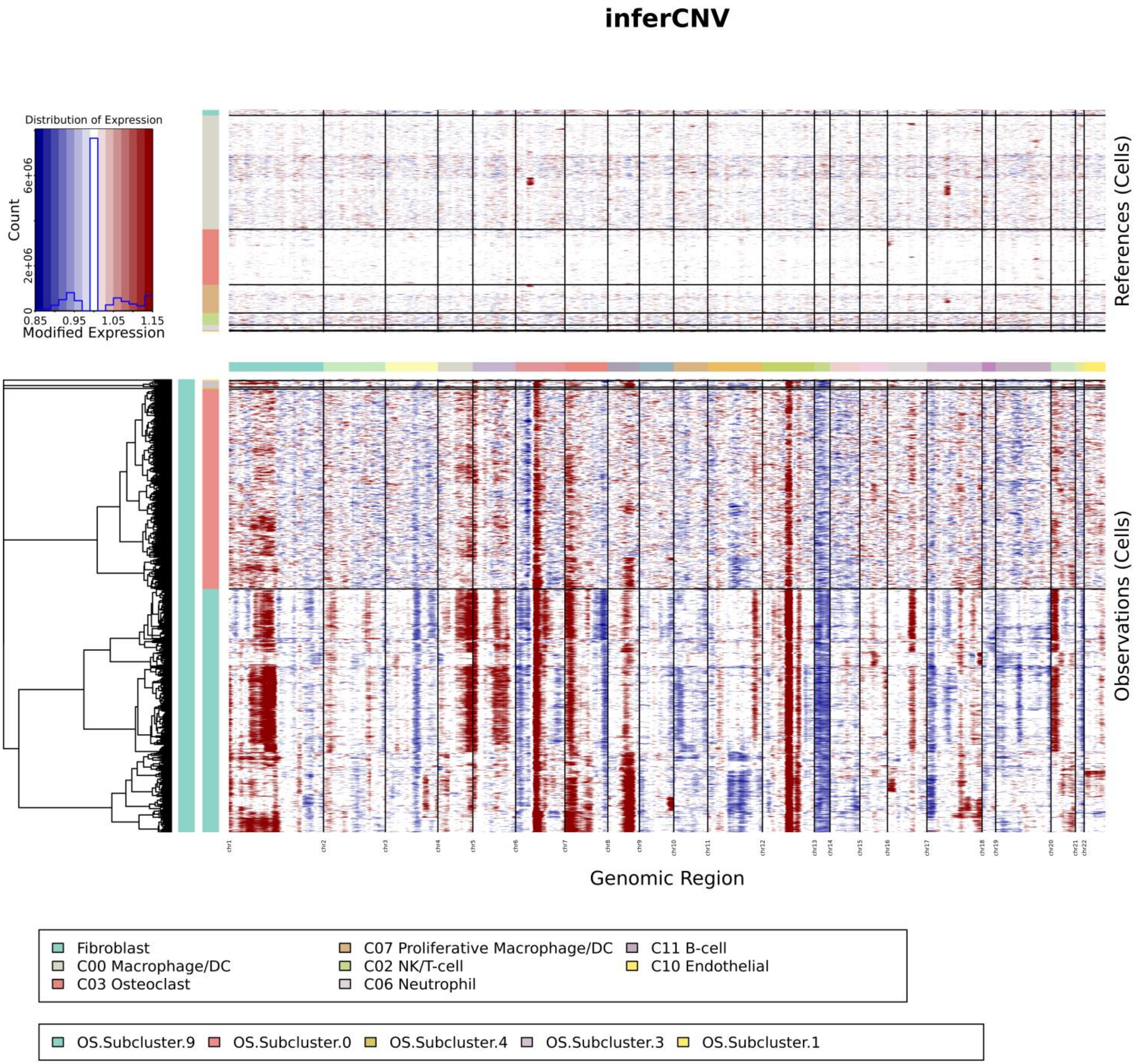
inferCNV
Distribution of Expression
Count
6e+06
2e+06
0
0.85
0.95
1.05
1.15
Modified Expression
References (Cells)
Observations (Cells)
chr1
chr2
chr3
chr4
chr5
chr6
chr7
chr8
chr9
chr10
chr11
chr12
chr13
chr14
chr15
chr16
chr17
chr18
chr19
chr20
chr21
chr22
Genomic Region
Fibroblast
C00 Macrophage/DC
C03 Osteoclast
C07 Proliferative Macrophage/DC
C02 NK/T-cell
C06 Neutrophil
C11 B-cell
C10 Endothelial
OS.Subcluster.9
OS.Subcluster.0
OS.Subcluster.4
OS.Subcluster.3
OS.Subcluster.1

OS3

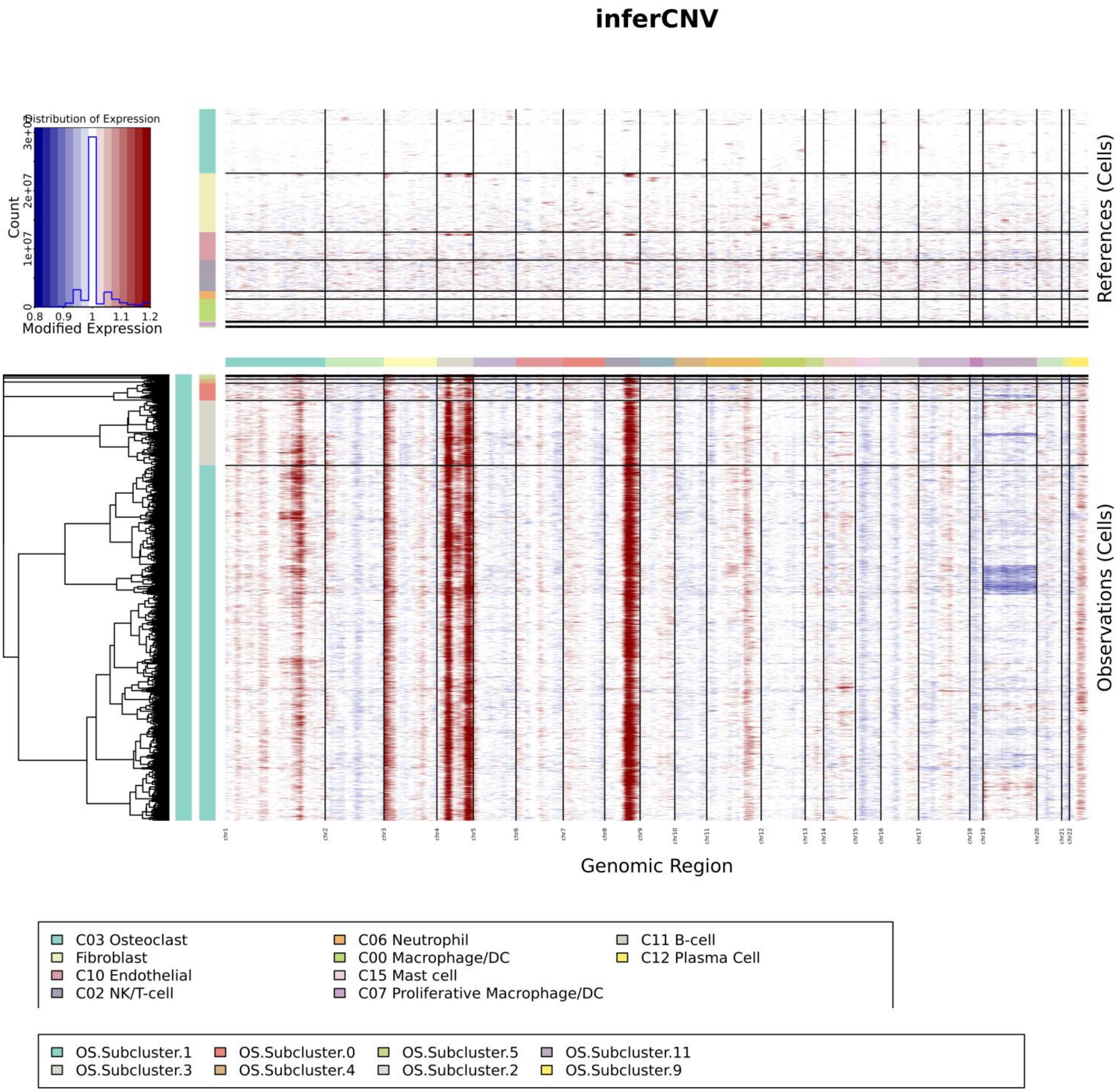
inferCNV
Distribution of Expression
Count
Modified Expression
References (Cells)
Observations (Cells)
Genomic Region
chr1 chr2 chr3 chr4 chr5 chr6 chr7 chr8 chr9 chr10 chr11 chr12 chr13 chr14 chr15 chr16 chr17 chr18 chr19 chr20 chr21 chr22
C03 Osteoclast
Fibroblast
C10 Endothelial
C02 NK/T-cell
C06 Neutrophil
C00 Macrophage/DC
C15 Mast cell
C07 Proliferative Macrophage/DC
C11 B-cell
C12 Plasma Cell
OS.Subcluster.1
OS.Subcluster.3
OS.Subcluster.0
OS.Subcluster.4
OS.Subcluster.5
OS.Subcluster.2
OS.Subcluster.11
OS.Subcluster.9

OS4

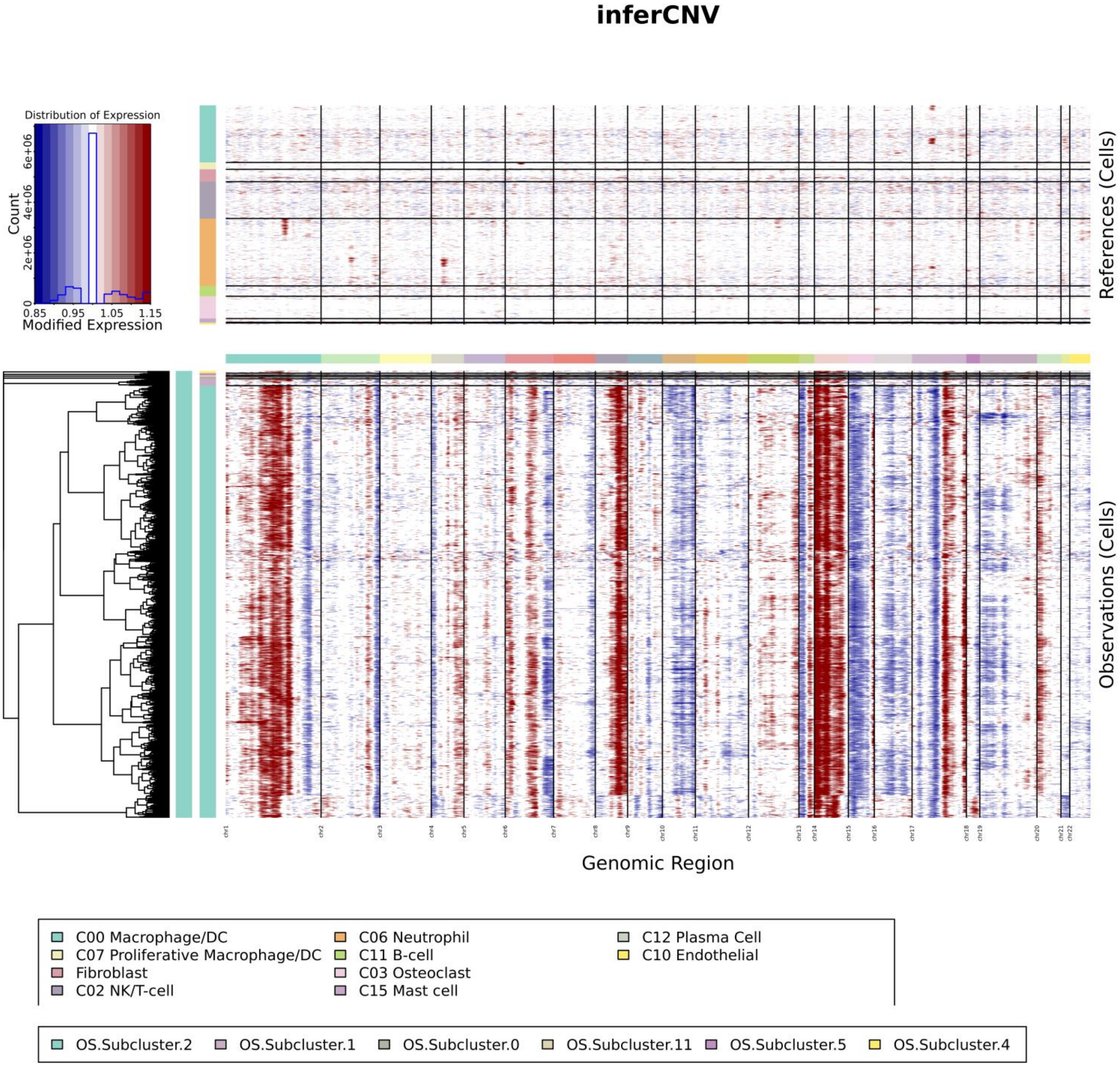

inferCNV
Distribution of Expression
Count
0
2e+06
4e+06
6e+06
0.85
0.95
1.05
1.15
Modified Expression
References (Cells)
Observations (Cells)
chr1
chr2
chr3
chr4
chr5
chr6
chr7
chr8
chr9
chr10
chr11
chr12
chr13
chr14
chr15
chr16
chr17
chr18
chr19
chr20
chr21
chr22
Genomic Region
C00 Macrophage/DC
C07 Proliferative Macrophage/DC
Fibroblast
C02 NK/T-cell
C06 Neutrophil
C11 B-cell
C03 Osteoclast
C15 Mast cell
C12 Plasma Cell
C10 Endothelial
OS.Subcluster.2
OS.Subcluster.1
OS.Subcluster.0
OS.Subcluster.11
OS.Subcluster.5
OS.Subcluster.4

OS5

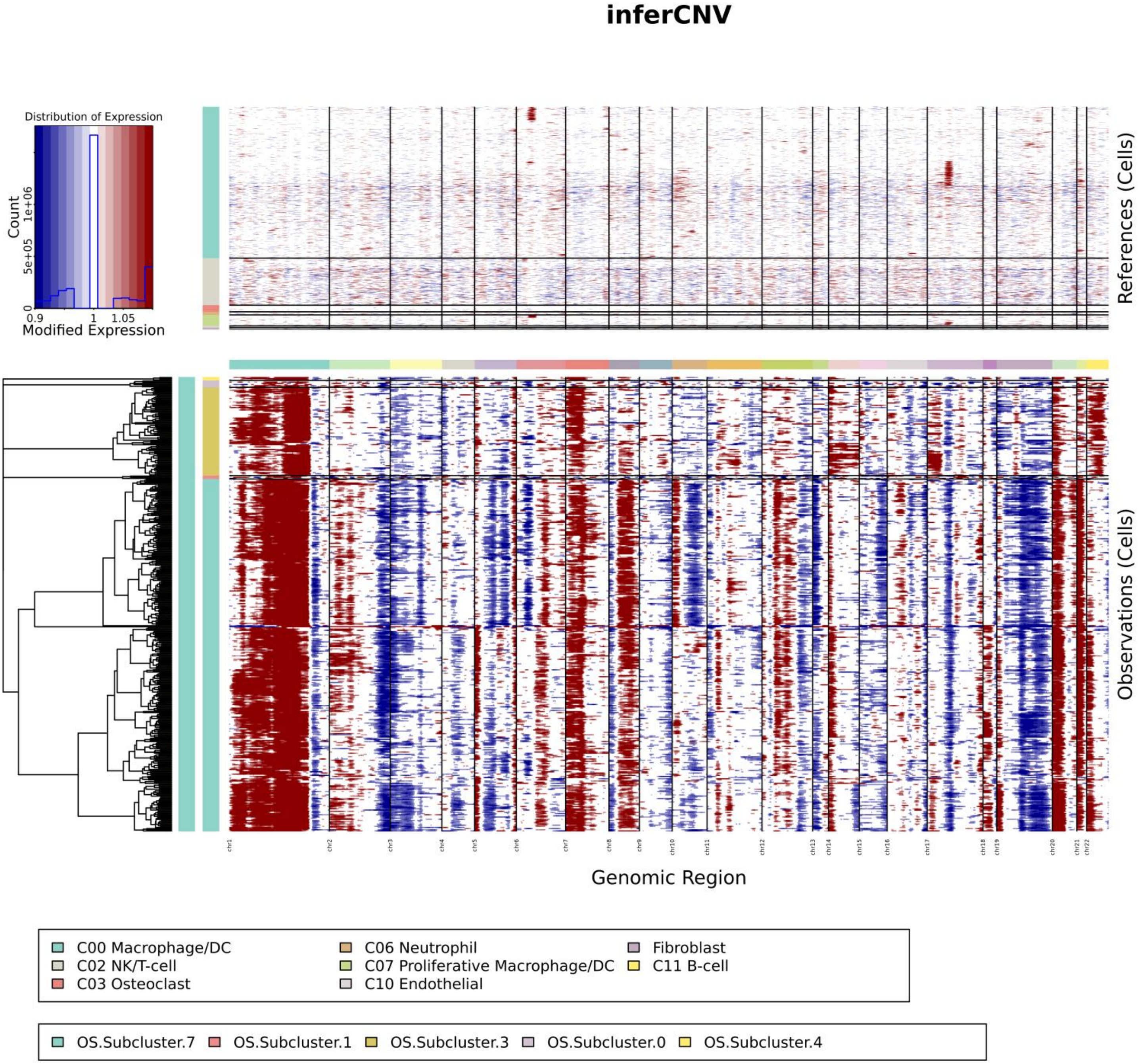
inferCNV
Distribution of Expression
Count
0
5e+05
1e+06
0.9
1
1.05
Modified Expression
References (Cells)
Observations (Cells)
chr1 chr2 chr3 chr4 chr5 chr6 chr7 chr8 chr9 chr10 chr11 chr12 chr13 chr14 chr15 chr16 chr17 chr18 chr19 chr20 chr21 chr22
Genomic Region
C00 Macrophage/DC
C02 NK/T-cell
C03 Osteoclast
C06 Neutrophil
C07 Proliferative Macrophage/DC
C10 Endothelial
Fibroblast
C11 B-cell
OS.Subcluster.7
OS.Subcluster.1
OS.Subcluster.3
OS.Subcluster.0
OS.Subcluster.4

OS6

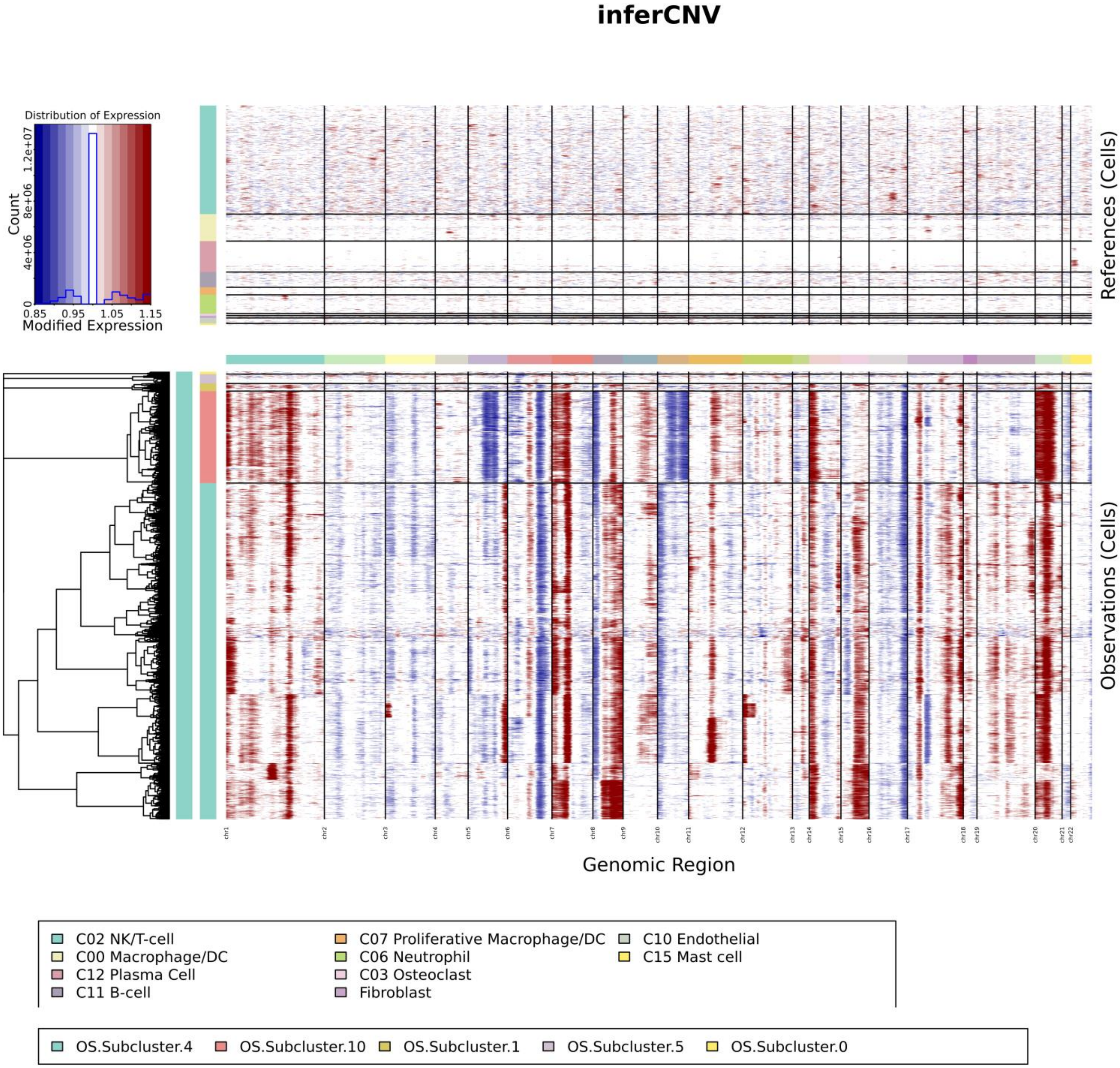
inferCNV
Distribution of Expression
Count
Modified Expression
0.85 0.95 1.05 1.15
0 4e+06 8e+06 1.2e+07
References (Cells)
Observations (Cells)
chr1 chr2 chr3 chr4 chr5 chr6 chr7 chr8 chr9 chr10 chr11 chr12 chr13 chr14 chr15 chr16 chr17 chr18 chr19 chr20 chr21 chr22
Genomic Region
C02 NK/T-cell
C00 Macrophage/DC
C12 Plasma Cell
C11 B-cell
C07 Proliferative Macrophage/DC
C06 Neutrophil
C03 Osteoclast
Fibroblast
C10 Endothelial
C15 Mast cell
OS.Subcluster.4
OS.Subcluster.10
OS.Subcluster.1
OS.Subcluster.5
OS.Subcluster.0
