# Supplementary figures and images for: Immunosuppressive Tumor Microenvironment of Osteosarcoma
Source: Cancers (Basel). 2025 Jun 24;17(13):2117. doi: 10.3390/cancers17132117 (PMC12248827; doi:10.3390/cancers17132117)

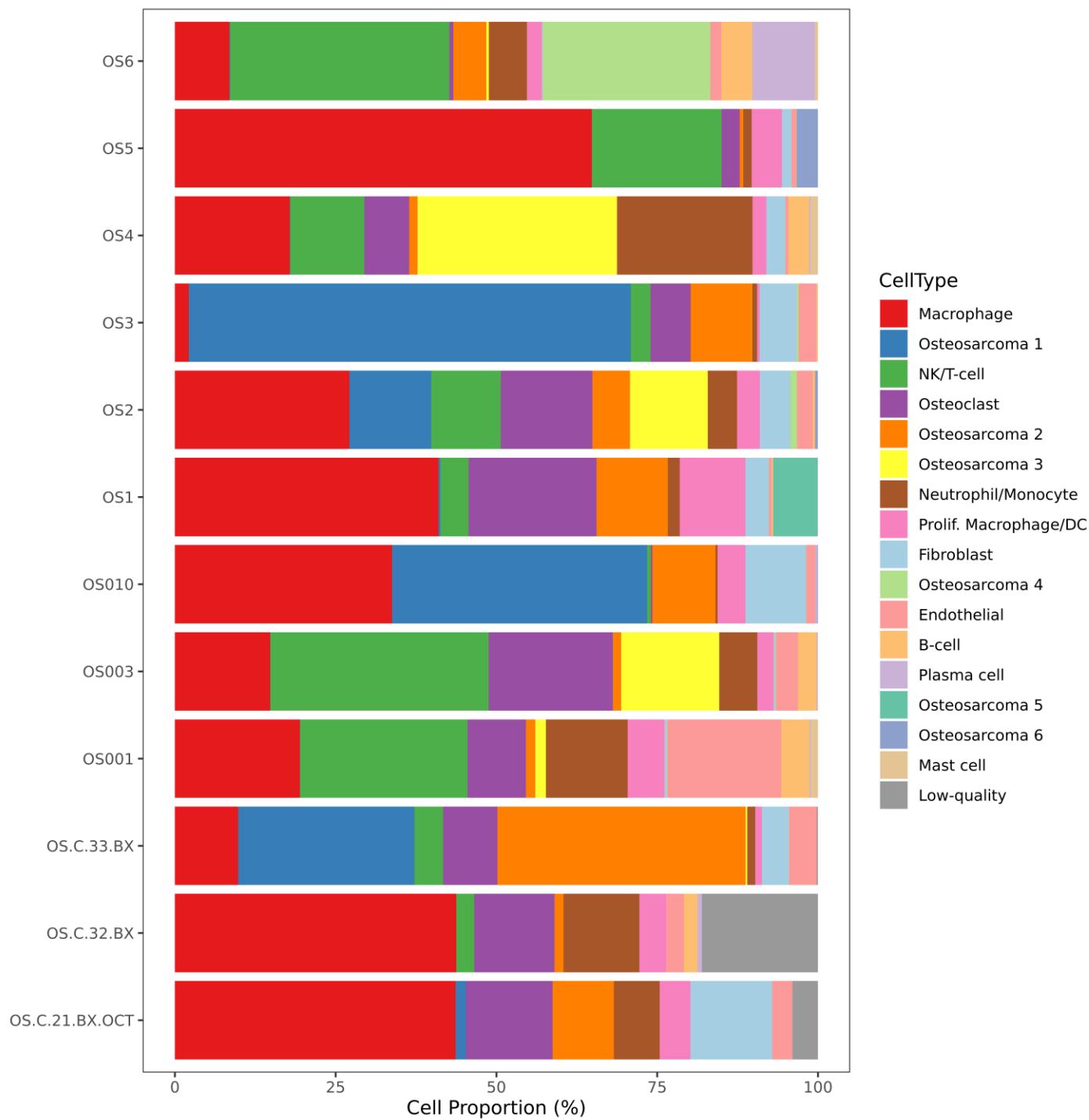

Supplement: Supplementary file 1 [file cancers-17-02117-s001.zip › Supplementary Figure S1 - Cell Proportions by Patient.pdf]

Ligand-Receptor

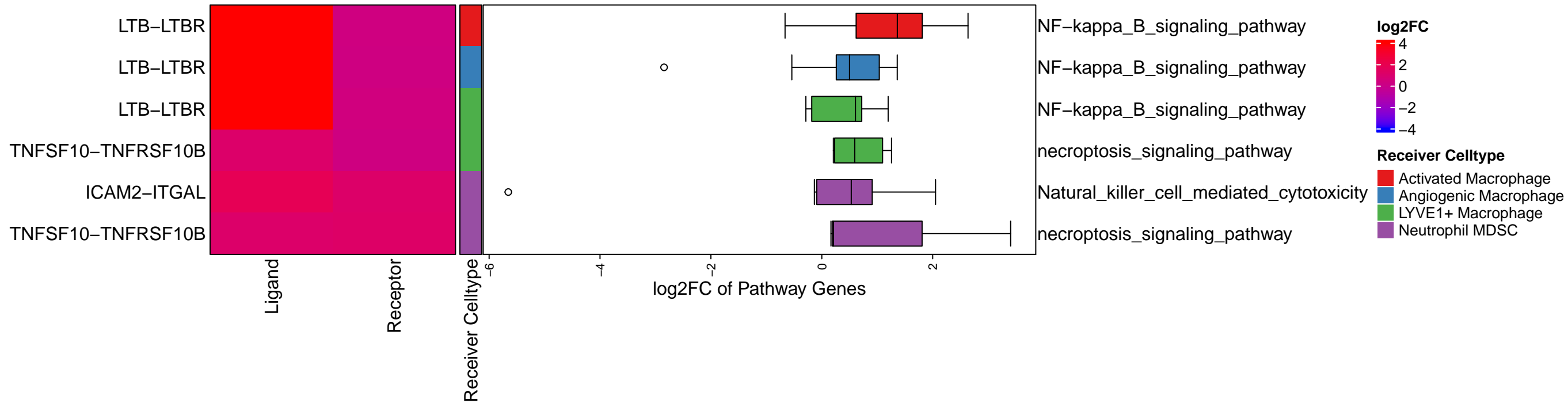

Supplement: Supplementary file 1 [file cancers-17-02117-s001.zip › Supplementary Figure S3 - Regulatory T-cell Ligand Interactions.pdf]

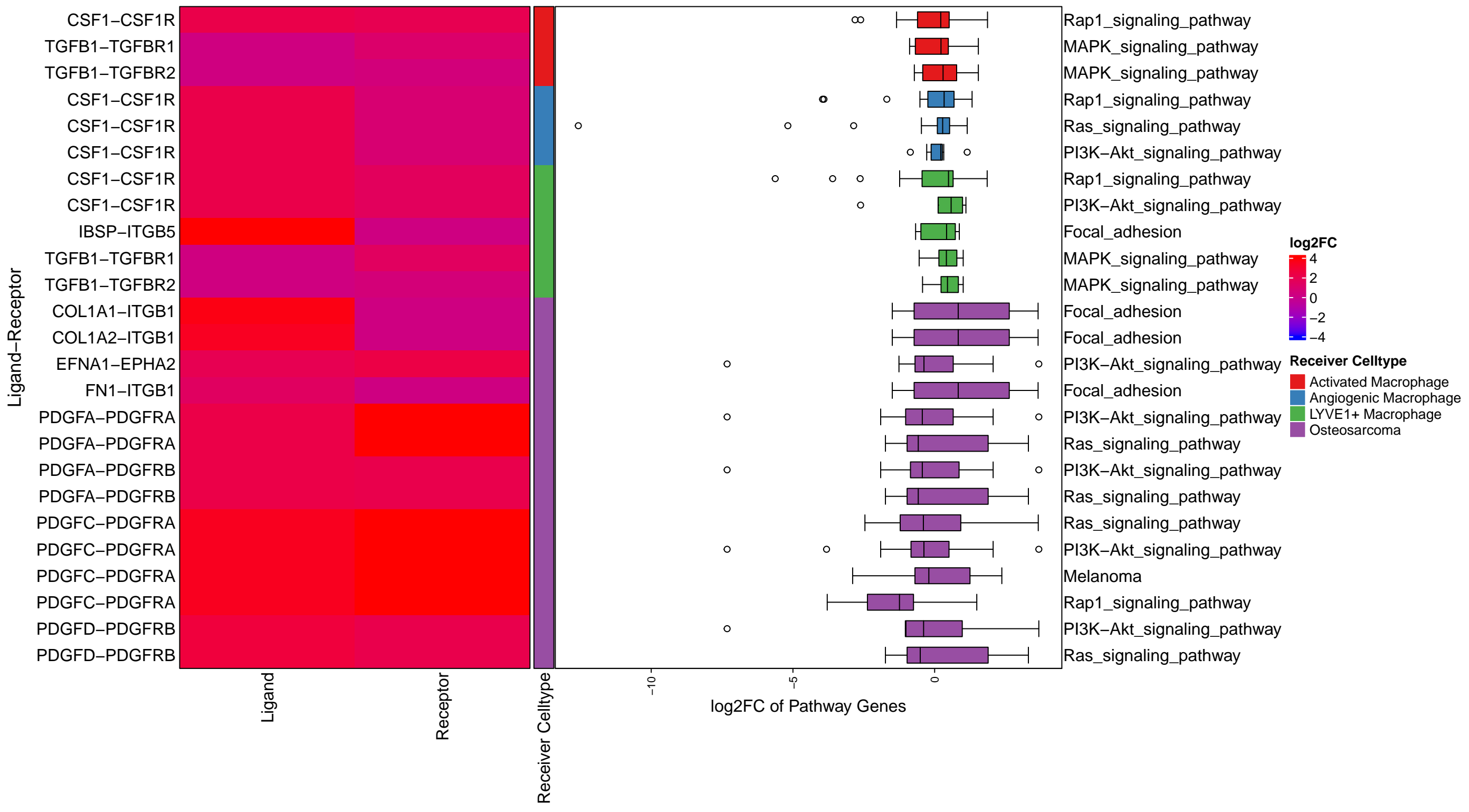

Supplement: Supplementary file 1 [file cancers-17-02117-s001.zip › Supplementary Figure S4 - Osteosarcoma Ligand Interactions.pdf]

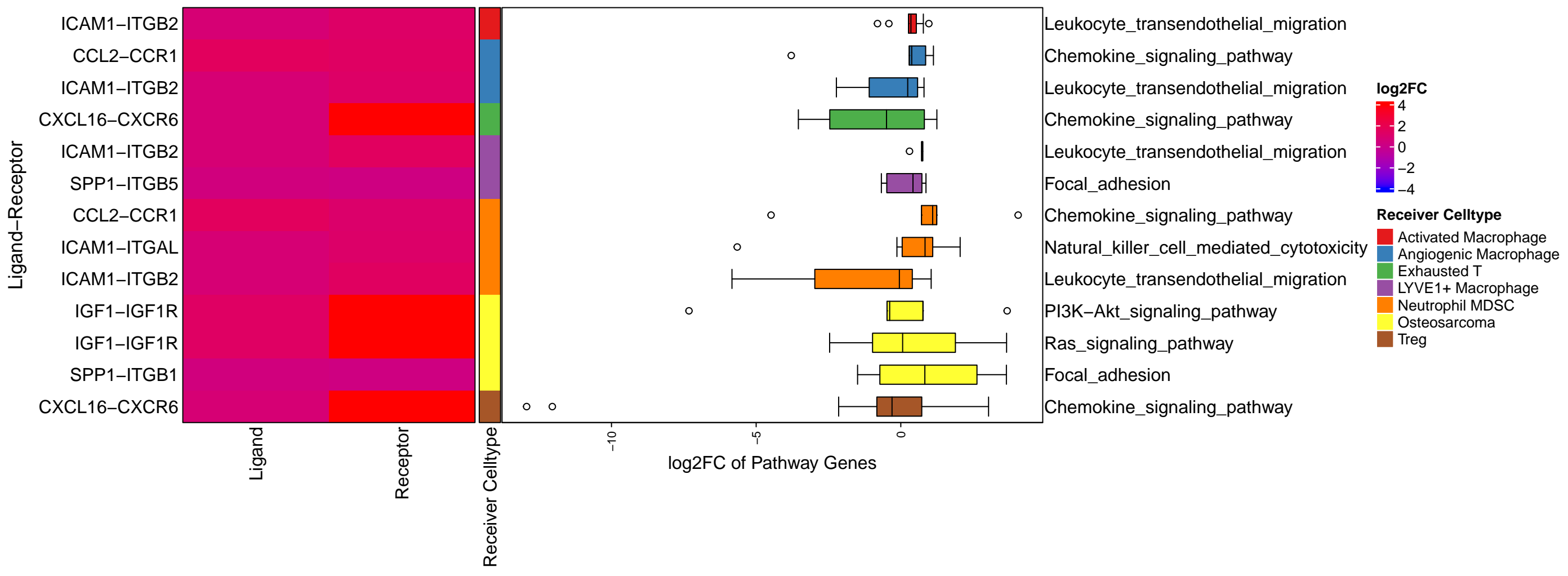

Supplement: Supplementary file 1 [file cancers-17-02117-s001.zip › Supplementary Figure S5 - LYVE1+ Macrophage Ligand Interactions.pdf]

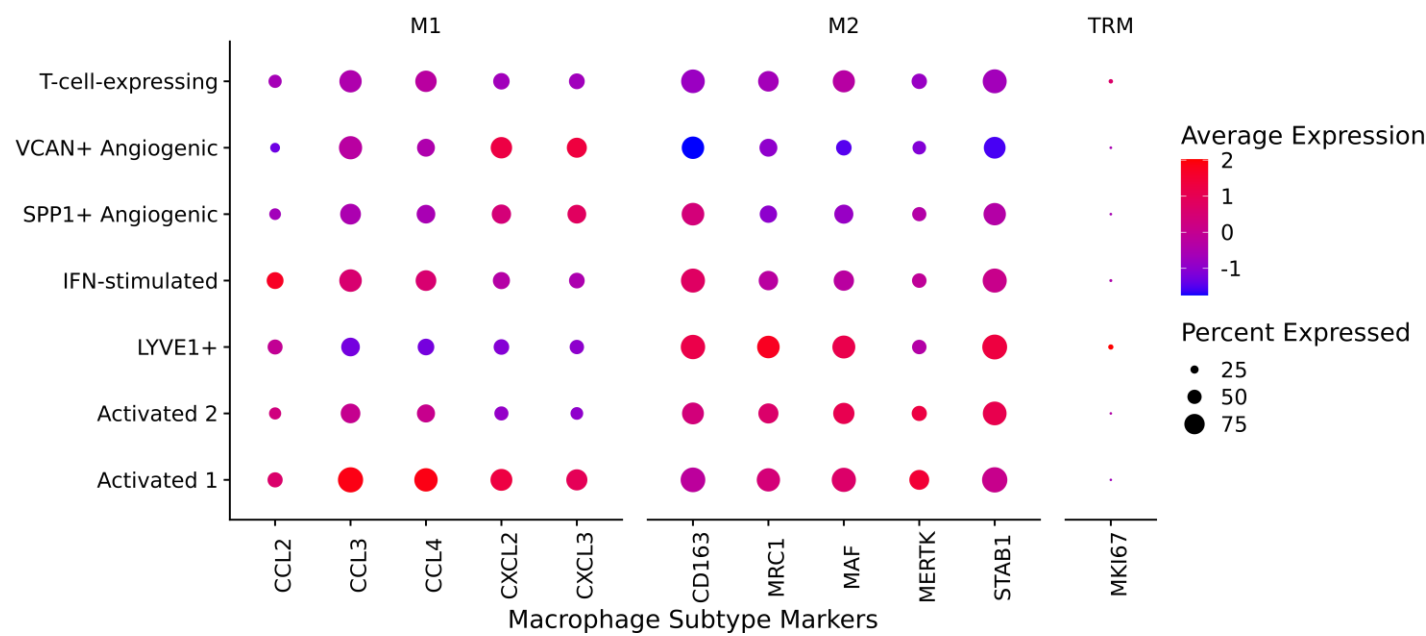

Supplement: Supplementary file 1 [file cancers-17-02117-s001.zip › Supplementary Figure S6 - Macrophage Subtype Markers.pdf]

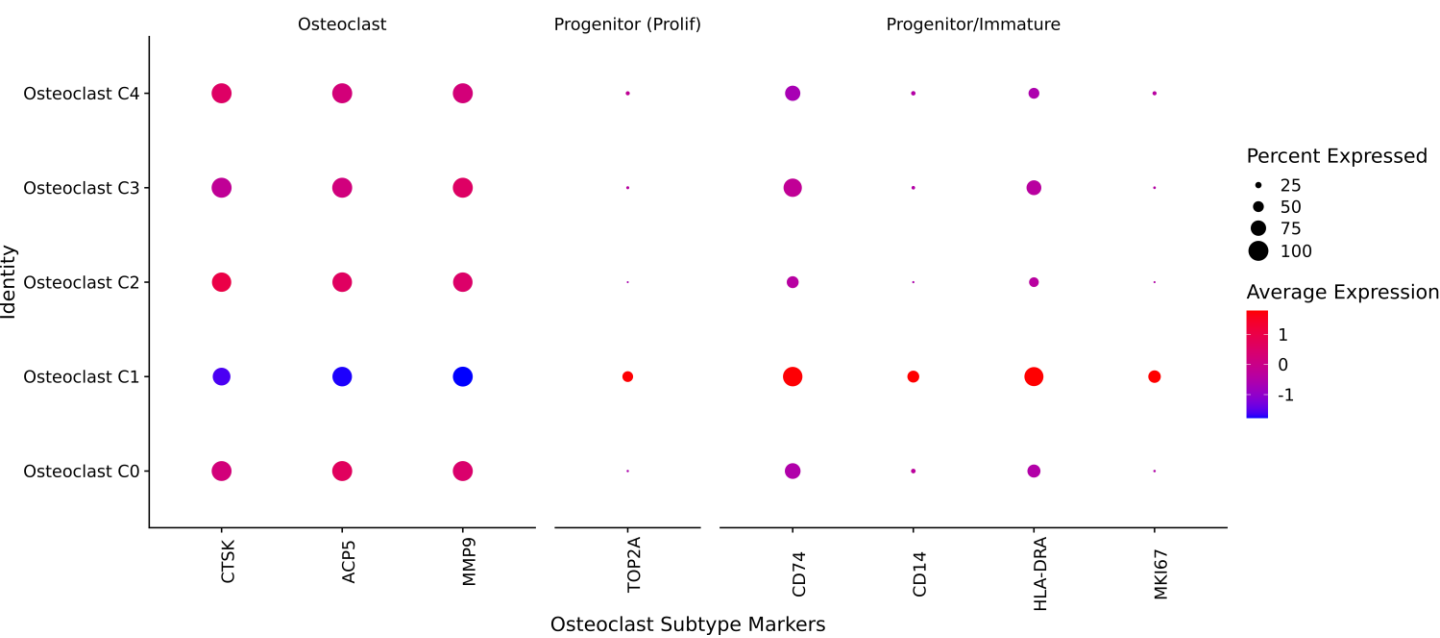

Supplement: Supplementary file 1 [file cancers-17-02117-s001.zip › Supplementary Figure S7 - Osteoclast Subtype Markers.pdf]
